# Supplementary figures and images for: Screening of Endophytic Fungi in Locoweed Induced by Heavy-Ion Irradiation and Study on Swainsonine Biosynthesis Pathway
Source: J Fungi (Basel). 2022 Sep 10;8(9):951. doi: 10.3390/jof8090951 (PMC9505577; doi:10.3390/jof8090951)

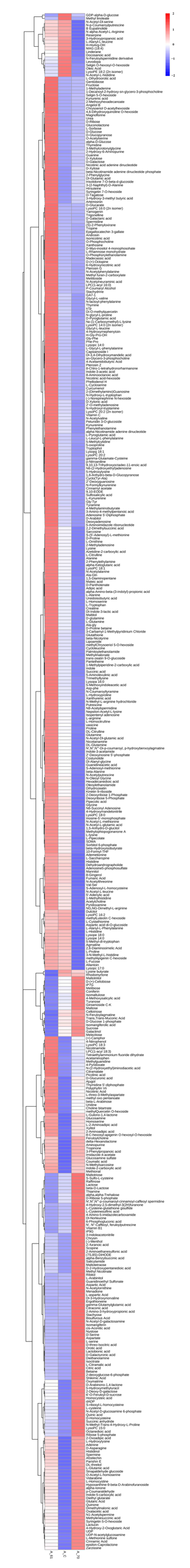

Supplement: Supplementary file 1 [file jof-08-00951-s001.zip › jof-1877879-supplementary Figure S1.tif]
